# Non‐profit breastfeeding organisations' peer support provision in areas of socio‐economic deprivation in the UK: A meta‐ethnography

**DOI:** 10.1111/mcn.13271

**Published:** 2021-09-30

**Authors:** Louise Hunt, Gill Thomson, Karen Whittaker, Fiona Dykes

**Affiliations:** ^1^ Maternal and Infant Nutrition and Nurture Unit (MAINN), School of Community Health and Midwifery University of Central Lancashire (UCLan) Preston UK; ^2^ MAINN, School of Community Health and Midwifery University of Central Lancashire (UCLan) Preston UK; ^3^ School of Nursing University of Central Lancashire (UCLan) Preston UK

**Keywords:** breastfeeding peer support, meta‐ethnography, non‐profit, peer support, socio‐economic deprivation, systematic review

## Abstract

In many high‐income countries such as the United Kingdom, inequalities in breastfeeding initiation and continuation rates exist, whereby socio‐economically advantaged mothers are most likely to breastfeed. Breastfeeding peer support interventions are recommended to address this inequality, with non‐profit breastfeeding organisations providing such support in areas of deprivation. As these organisations' roots and membership are often formed of relatively highly resourced women who have different backgrounds and experiences to those living in areas of deprivation, it is important to understand their practices in this context. In order to explore how UK non‐profit organisations practice breastfeeding peer support in areas of socio‐economic deprivation, a systematic review and meta‐ethnography of published and grey literature was undertaken. Sixteen texts were included, and three core themes constructed: (1) ‘changing communities’ reveals practices designed to generate community level change, and (2) ‘enabling one to one support’, explains how proactive working practices enabled individual mothers' access to supportive environments. (3) ‘forging partnerships with health professionals’, describes how embedding peer support within local health services facilitated peer supporters' access to mothers. While few breastfeeding peer support practices were directly linked to the context of socio‐economic deprivation, those described sought to influence community and individual level change. They illuminate the importance of interprofessional working. Further work to consolidate the peer‐professional interface to ensure needs‐led care is required.

**Key messages**

- Organisational practices aimed to influence community change and meet individual mothers' needs through proactive contacts.
- Practices highlight the importance of embedding breastfeeding peer support with local health services to facilitate peer supporter's access to women.
- Future studies should adopt a context‐based approach, include the views of mothers who do not engage with services and actively target those who are socio‐economically deprived.

## 1 INTRODUCTION

The health gains of breastfeeding in high, middle‐ and low‐income countries are indisputable (Grummer‐Strawn & Rollins, 2015), yet rates remain below recommendations internationally (WHO, 2019). In the United Kingdom, highly educated women living in the least deprived areas have the highest incidence of breastfeeding, while mothers living in areas of socio‐economic deprivation are least likely to breastfeed (McAndrew et al., 2012; Public Health England [PHE], 2020), a prevalence pattern repeated across high income countries (UNICEF, 2018). Peer support has been defined as:

The provision of emotional, appraisal, and informational assistance by a created social network member who possesses experiential knowledge of a specific behaviour or stressor and similar characteristics as the target population (Dennis, 2003, p. 329).

Breastfeeding peer support (BPS) interventions are nationally and internationally recommended to increase breastfeeding rates (NICE, 2008; WHO, 2003) and address inequalities (DH & DCSF, 2009; PHE & UNICEF, 2016). However, when results of BPS trials in high‐income countries (particularly the United Kingdom) are aggregated, they have been found to be ineffective in increasing breastfeeding rates (Jolly et al., 2012), although two recent trials of pro‐active peer support have had positive outcomes (Clarke et al., 2020; Forster et al., 2019). Qualitative research highlights peer support's value in promoting breastfeeding continuation and maternal well‐being (e.g., Thomson, Crossland, & Dykes, 2012), and additional support from lay supporters and professionals has been found to effect breastfeeding outcomes positively (McFadden et al., 2017). Despite this mixed evidence, UK commissioning of BPS continues, and it is provided in 56% of areas (Grant et al., 2018). Non‐profit organisations make money for social purposes or provide services people need (Cambridge Dictionary, 2020), comprising faith and community groups, social enterprises and charities and their activities take place between the market, state and family (Rees & Mullins, 2017). Non‐profit organisations are involved in supporting breastfeeding mothers in a range of high‐income countries, for example, Australia (i.e., McLardie‐Hore et al., 2020), Ireland (i.e., McCarthy Quinn et al., 2019) and the United Kingdom where government policy has encouraged their involvement in health services generally, and as part of efforts to impact health inequalities (Voluntary, Community and Social Enterprise Review [VCSE], 2018). UK national non‐profit breastfeeding organisations have been commissioned to provide BPS interventions in areas of deprivation, yet their roots and membership are formed of relatively wealthy women (i.e., La Leche League, 2020). Peer support is grounded on the belief that people learn more effectively from peers with whom they identify and share common experiences (Dennis, 2003). However, insights into how UK national non‐profit breastfeeding organisations adapt services for areas of deprivation are lacking as qualitative accounts have yet to be synthesised. Understanding organisational practice in this context will enable better service design and has implications for breastfeeding peer supporters (PSs) and other health care professionals in the United Kingdom and elsewhere. This paper presents a meta‐ethnography of existing qualitative research to address the review question: How do UK national non‐profit breastfeeding organisations practice BPS in areas of deprivation?

## 2 METHODOLOGY

Meta‐ethnography is widely used in health research (France et al., 2019), and, as our focus lay with organisational practices in context rather than on outcomes, was considered more useful than an integrative review including quantitative data. Noblit and Hare's (1988) meta‐ethnographic approach was selected for its logical steps and congruence with our interpretive theoretical position. The eMERGe guidance developed to clarify meta‐ethnographic reporting is used to report this review (France et al., 2019).

### 2.1 Search strategy

Our search strategy aimed to identify all qualitative accounts of UK non‐profit breastfeeding organisations' practices in areas of deprivation. We used a predetermined search strategy, a quality appraisal tool (Downe et al., 2009) and meta‐ethnographic data analysis techniques (Noblit & Hare, 1988). The population, exposure and outcomes (PEO) framework was used to develop the review (see Table 1). Search terms were formulated by testing them across databases, and term truncations adapted for different databases (see Table 1). Four databases were searched (Embase, PsycINFO, CINAHL and MEDLINE with full text). To identify grey literature, established search strategies were followed (i.e., Godin et al., 2015). This involved contacting UK national non‐profit breastfeeding organisations for suggestions, culminating in a list of websites (see Data S1) that were searched via key terms or hand searched as appropriate. All studies meeting inclusion criteria were subjected to backward and forward chaining, journal run (hand searching relevant journals, e.g., *Maternal and Child Nutrition*), and a key author search.

**Table 1:** PEO approach and search terms

Criteria	Inclusion criteria	Associated search terms
Population	UK women living in areas of socio‐economic deprivation.	women (woman, maternal, mother, patient, consumer, service user), socio‐economic deprivation (socioeconomic, deprivation, marginalisation, disadvantage, low income, poverty, inequality, poorest, underprivileged, vulnerable),
Exposure	BPS practices and interventions provided by UK national non‐profit breastfeeding organisations	peer support (peer support, lay support, volunteer support, mother to mother, counsellor, non‐professional, volunteer, peer group, lay, voluntary worker),
Outcome	Breastfeeding	Breastfeeding (breastfeeding, breastfed, infant feeding, lactation, milk human, nursing mother).

### 2.2 Search processes

LH carried out the searches (time restrictions precluded two researchers undertaking this task). To capture all studies, no date restriction was applied and all qualitative study types included (i.e., ethnographic, grounded theory, narrative, phenomenological, case studies or mixed‐methods studies with a clear qualitative component). As we sought UK studies, only English language papers were included. Deprivation can be measured in multiple ways (Galobardes et al., 2007). We included any study which deliberately attempted to work in an area of deprivation defined by any measure. For example, some studies defined areas by income (Curtis et al., 2007), others by area‐based deprivation measures (Thomson et al., 2015). We included any study reporting a project involving a UK national non‐profit organisation, defining involvement as; the organisation or its representatives having run the project or trained and/or provided ongoing support for PSs.

### 2.3 Selecting primary studies

LH undertook screening and selection, with decision making reviewed by other team members. Screening comprised papers being screened by title and abstract, and full‐text papers being reviewed against inclusion criteria. All texts deemed relevant were quality assessed using an appraisal tool (Downe et al., 2009) that assessed key study features and assigned a score from A to D (see Table 2). All studies graded A were included, those graded D excluded. Those graded B and C were discussed within the team, and a list of included studies agreed. Data were extracted by repeated reading of full texts, during which research designs, aims, focus, theoretical approaches and study dates were extracted and compared. Studies were uploaded onto MAXQDA software and findings sections coded. In line with our focus on practice in context, we did not aim to include all practices. Many papers discussed practices common to all contexts. However, for the purposes of this review, we only focused on practices that were specifically related to the context of deprivation.

**Table 2:** Study characteristics and quality appraisal rating (*n* = 16)

Author Year	QA grade	Aim	Study design	Location and participant characteristics (age, SES, ethnicity)	Number of participants	Data collection methods	Analysis methods	Whether associated with another study	Non‐profit organisation involved
Raine (2003)	C	Evaluation	Qualitative evaluation	Northern England. PSs aged 24–41, married, with at least one child	Health professionals (*n* = 6); PSs (*n* = 6); mothers (*n* = 6). 7 PSs also kept diaries	Semi structured interviews, observations, PSs diaries	Grounded theory	Same project as Raine and Woodward (2003). Also in Dykes (2005)	LLL
Raine and Woodward (2003)	C	Evaluation	As above	As above	As above	As above	As above	Same project as Raine (2003).	LLL
Battersby (2001)	C	Evaluation	Qualitative evaluation	Northern England	Mothers (*n* = 16)	Interviews, questionnaire	Not stated	Also in Dykes (2005)	LLL
Dykes (2005)	B	Evaluate government projects.	Qualitative evaluation	All projects in areas of deprivation.	Not stated	Project summaries thematically assessed.	Thematic analysis	Includes Raine (2003); Raine and Woodward (2003); Battersby, (2001); Kirkham et al. (2006); Curtis et al. (2007)	Does not stipulate which organisation involved with which project
Curtis et al. (2007)	A	Explore peer–professional interface	Descriptive qualitative study	PSs from, and health professionals worked in,’in need’ areas	PS (*n* = 7); community health professionals (*n* = 9)	Focus groups	Thematic analysis	Same project as Kirkham et al. (2006). Also in Dykes (2005)	NCT
Kirkham et al. (2006)	C	Report project	Narrative report	Northern England	NA	Personal reports, quotations from project reports, other academic writing	Narrative report	Same project as Curtis et al. (2007). Also in Dykes (2005)	NCT
Graffy and Taylor (2005)	A	Explore support women want	Questionnaire part of trial	London. Range of ages and SES but 38% of mothers had partners with highest banded managerial jobs. 13% had partners with lowest banded jobs. Range of ethnicity but 68% white, 16% African/Caribbean	Mothers (*n* = 654)	Questionnaire with open questions	Thematic grounded theory	No	NCT
Ingram et al. (2005)	A	Evaluation	Mixed methods evaluation	Southern England. Mothers lived in high deprivation areas	PS (*n* = 6); Mothers (*n* = 35)	Questionnaires (mothers & PSs), focus groups (PSs); Breastfeeding rate analysis.	Thematic analysis of focus group	No	LLL and ABM
Ingram (2013)	A	Evaluation	As above	Southern England. Mothers aged 16–40	Mothers' surveys (*n* = 163) and interviews (*n* = 14). Health professional interviews (*n* = 8). PS focus group (*n* = 7)	Online surveys, telephone interviews, focus groups	Inductive thematic analysis of interviews. Mixed methods data triangulation	No	As above
South et al. (2012)	A	Report user perspectives	Multiple case study (BPS one case)	Northern England. Some mothers and one PS from minority ethnic communities	Mother interview (*n* = 1); Mother focus group (*n* = 10)	Interview, focus group	Qualitative thematic analysis, cross case analysis	No	LLL
Fox et al. (2015)	A	Explore user experiences	Qualitative evaluation	Eight sites in areas of deprivation. North to South England, rural and urban. Age:23–44 years. SES: Not given but most highly educated living with partner. Range of ethnicities	Mothers (*n* = 51)	As above	Inductive qualitative analysis	No	NCT
Crossland and Thomson (2013)	A	Explore health professional perspectives	Action‐based qualitative evaluation	Northern England	Health professionals (*n* = 40)	Group and or individual interviews	Thematic analysis	Same project as Aiken and Thomson (2013) and Thomson, Crossland, and Dykes (2012)	BfN
Aiken and Thomson (2013)	A	Discuss professionalisation	As above	Northern England	PSs (*n* = 19)	As above	As above	Same project as Crossland and Thomson (2013) and Thomson, Crossland, and Dykes (2012)	BfN
Thomson, Crossland, and Dykes (2012)	A	Explore practice–theory interface	As above	Northern England. Mothers aged 19–39. Marital status: Single (*n* = 5), Partner (*n* = 14), Married (*n* = 28)	Mothers (*n* = 47)	Individual interview	Qualitative data analysis using a theoretical framework of hope	Same project as Aiken and Thomson (2013) and Crossland and Thomson (2013)	BfN
Thomson, Dykes, et al. (2012)	A	Explore incentive project	Qualitative evaluation	Northern England. Mothers aged 21–42. Ethnicity: Asian (*n* = 1) White British (*n* = 25)	Mothers (*n* = 26); PS (*n* = 4)	Mother individual interviews; PSs focus group	Qualitative thematic analysis and project data descriptive analysis	No	BfN
Thomson et al. (2015)	A	Evaluation	Qualitative evaluation	Northern England. Mixed area but several wards highly deprived. Mothers aged 19–47. Ethnicity: White British (*n* = 23), Latin American (*n* = 1)	Mothers (*n* = 24); PS (*n* = 13); health professionals (*n* = 50)	Individual and group interviews. Project data evaluation	Thematic analysis using social capital lens	No	BfN

### 2.4 Data synthesis strategy

LH undertook initial data synthesis with decisions shared and discussed with all other members of the research team. Noblit and Hare's (1988) inductive, interpretive meta‐ethnographic method was used to analyse data. This approach differentiates between first‐, second‐ and third‐order data (Toye et al., 2014). First‐order concerns participant quotes, second‐order, paper authors' interpretations, and third‐order, new interpretations generated by the review team (Toye et al., 2014). Meta‐ethnography involves working with second‐order data to ‘translate studies into one another’ (Noblit & Hare, 1988). This process uses the constant comparative method (Charmaz, 2006) to note differences and similarities between second‐order concepts and group them into conceptual categories creating third‐order interpretations (Noblit & Hare, 1988). First‐order data (quotes) are selected to substantiate interpretations generated. The different forms of translation concern reciprocal (identifying what was similar), refutational (identifying disconfirming data) and a line of argument synthesis (an overarching synthesis) (Noblit & Hare, 1988).

## 3 RESULTS

**Figure 1:**
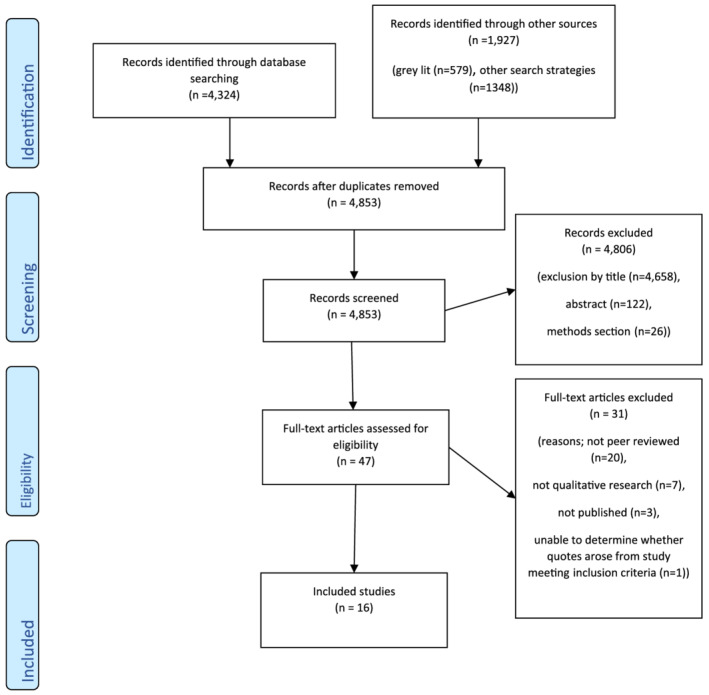
PRISMA diagram

The search, dated June 2020, identified 4,324 records via database searches, and 1,927 via grey literature and other search strategies (described above). Once duplicates were removed, 4,853 records were screened and 4,806 excluded. Quality assessment was applied to the 47 remaining records, and a further 31 excluded (these arose from grey literature), leaving 16 included studies (Figure 1, PRISMA diagram).

Table two shows study characteristics and quality assessment grades. Studies include the views of 1,033 mothers, 62 PSs and 113 health professionals. Mothers' ages ranged from 16 to 47 years with most studies including those with a range of parities and feeding experiences (i.e., women with experience of breastfeeding for a short time, long time, mixed feeding and feeding expressed breast milk). Most included studies focused more on the process of setting up and running a BPS project and the experiences of those involved, than on breastfeeding outcomes. Many studies included the perspectives of women, peer supporters and health professionals. Studies report on 35 projects (some report the same project. See Table 2) which involve La Leche League (LLL) (*n* = 6), National Childbirth Trust (NCT) (*n* = 5), Breastfeeding Network (BfN) (*n* = 4) and the Association of Breastfeeding Mothers (ABM) (*n* = 2). The numbers do not total 35 as Dykes (2005) reports on 26 projects but does not detail specific organisational involvement, and two studies (Ingram, 2013; Ingram et al., 2005) report projects involving two organisations. Although all studies took place in areas of deprivation, only one reported participants' socio‐economic characteristics (Graffy & Taylor, 2005). Other studies reported area level measures of deprivation. Seven studies reported projects primarily focused on community change, eight on individual change measured by breastfeeding rates, and one on both. While most studies were qualitative evaluations, one formed the qualitative part of a randomised controlled trial, two were mixed methods evaluations, and one a multiple case study. Most studies employed interviews and focus groups, although questionnaires, observations and diary keeping methods were also used. Most studies analysed findings thematically.

Data analysis identified 28 second‐order concepts. Translational synthesis was reciprocal; although studies reported differing practices, reasons for using each practice did not conflict. Three core third‐order themes and their associated subthemes were constructed (see Table 3) and are discussed below using first‐order quotes.

**Table 3:** Core and subtheme associations

Core themes	‘Changing communities’		‘Enabling one‐to‐one support’			‘Forging partnerships with health professionals’	
Subthemes	‘Getting the message out’	‘Enabling supportive social contact’	‘Building support networks’	‘Being proactive’	‘Enabling needs‐led care’	‘Building trust’	‘Collaborating via communication’
Raine (2003)	X	X	X			X	
Raine and Woodward (2003)	X	X				X	
Battersby (2001)	X					X	
Dykes (2005)	X	X				X	X
Curtis et al. (2007)	X	X				X	
Kirkham et al. (2006)	X	X				X	X
Graffy and Taylor (2005)							
Ingram et al. (2005)		X	X				
Ingram (2013)		X	X	X		X	X
South et al. (2012)		X			X	X	
Fox et al. (2015)		X	X				
Crossland and Thomson (2013)				X		X	
Aiken and Thomson (2013)				X		X	X
Thomson, Crossland, and Dykes (2012)		X	X	X	X		
Thomson, Dykes, et al. (2012)		X	X	X	X	X	X
Thomson et al. (2015)	X	X	X	X	X	X	X

### 3.1 Theme 1 ‘Changing communities’

This theme focuses upon how projects sought to generate community change through breastfeeding promotion practices (‘getting the message out’) and using groups to provide supportive social environments for breastfeeding mothers (‘enabling supportive social contact’).

Several studies described communities where breastfeeding was little seen or spoken of, where breastfeeding knowledge, skills, and traditions had been lost (Battersby, 2001; Curtis et al., 2007; Dykes, 2005; Kirkham et al., 2006; Raine, 2003; Raine & Woodward, 2003; South et al., 2012; Thomson et al., 2015). For example, a project coordinator in Raine's (2003) study reported:

The community has no knowledge generally of breastfeeding, and although it might seem that it should just be something that mothers would know, it isn't at all (p. 646).

Some studies reported communities with few informal support networks (e.g., friendship networks forged via clubs or associations), leaving some women isolated from other breastfeeding women (Fox et al., 2015; Ingram et al., 2005; Kirkham et al., 2006; Raine, 2003; Raine & Woodward, 2003; Thomson, Crossland, & Dykes, 2012):

At the time I felt like I was the only one breastfeeding. You don't realise there's lots of other people around you cause it's not something you talk about every day (breastfeeding mother) (Raine & Woodward, 2003, p. 212).

‘Getting the message out’ refers to practices employed to make breastfeeding more visible and acceptable in communities. On a personal level, some PSs wanted to use every opportunity to extol the benefits of breastfeeding (Battersby, 2001; Curtis et al., 2007; Ingram et al., 2005; Raine & Woodward, 2003). However, Raine and Woodward (2003) explain the delicate line between this, and ensuring they understood the situations of individual women who might not have continued to breastfeed:

Sometimes I think they just need to understand that not everyone can (breast)feed. Cause all they seem to go an about is breastfeeding…. (breastfeeding mother, p. 213).

Breastfeeding was promoted using local media (Thomson et al., 2015), by introducing breastfeeding friendly café and town schemes (Raine, 2003;Raine & Woodward, 2003; Thomson et al., 2015), educational work in schools (Kirkham et al., 2006; Thomson et al., 2015) and organising and participating in community events (Raine & Woodward, 2003; Thomson et al., 2015). PSs became ‘known’ and ‘visible’ by identifying themselves and their roles while on and off duty (Curtis et al., 2007; Thomson et al., 2015). For example, wearing their uniform (a T‐shirt displaying service details) to personal healthcare appointments:

I saw my doctor as a personal thing for me and she said, ‘Oh you do something around breastfeeding don't you?’ So, I don't know whether, again, that makes any difference in her other role, but maybe a mum goes to her and says, oh I'm finding it hard and she might go, oh well I know that there's a group. (PS_6) (Thomson et al., 2015, p. 9).

These activities were felt to form ripples of influence, designed to normalise breastfeeding within the community (Dykes, 2005; Ingram et al., 2005; Raine & Woodward, 2003; Thomson et al., 2015).

‘Enabling supportive social contact’ describes how breastfeeding groups were often used to foster social support, and outlines the importance of facilitating mothers' access to groups.

All projects, except those of Battersby (2001) and Graffy and Taylor (2005), used breastfeeding groups as part of their services. Although Kirkham et al. (2006) perceived groups as a means to deprofessionalise breastfeeding, all studies using groups emphasised their social value in creating opportunities for new friendships and as places where vicarious breastfeeding knowledge could be found:

I do think it's quite important, that here, you can speak to other mums with older babies and see it does get better, because if you're all sat here with newborns, all crying, all saying you can't do it. You want to see that it will get better, to speak to a mum that says its better (Mother, age 30, first baby) (Fox et al., 2015, p. 10).

Group attendance across studies was variable. One study reported poor attendance (Ingram, 2013), others, high (Ingram et al., 2005; Thomson et al., 2015). In the study by Ingram (2013), personal relationships were central to understanding attendance patterns:

Some mothers prefer to see their own peer supporter when they go to a group and are a bit reluctant to go if we are not going to be there. (PS #4) (p. 7).

In the incentive intervention when PSs–women relationships were strengthened by increased face to face contact, group attendance increased (Thomson, Dykes, et al., 2012). Conversely, when women must initiate attendance independently, some described feeling apprehensive (Fox et al., 2015; Ingram, 2013). Groups had the potential to be attended more readily by socially advantaged mothers compared to those younger, less confident, or less affluent (Fox et al., 2015).

### 3.2 Theme 2 ‘Enabling individual level support’

While individual level support includes enabling social contact as discussed above, this theme describes strategies to facilitate individual change such as harnessing support from mother's families (‘building support networks’), using pro‐active support (‘being pro‐active’), and building trust in PS‐mother relationships to facilitate needs‐led care (‘enabling needs‐led care’).

‘Building support networks’: As explained above, many study communities lacked breastfeeding knowledge. In several studies, to help enable women to meet their breastfeeding goals, PSs sought to strengthen their family support systems by involving partners and family members at every contact (Ingram, 2013; Thomson et al., 2015;Thomson, Crossland, & Dykes, 2012; Thomson, Dykes, et al., 2012), opportunistically enabling family members to access support via increased community visibility (Thomson et al., 2015), running grandmother peer support training (Thomson et al., 2015) and making partners and family members welcome at breastfeeding groups:

We thought fathers were not allowed to stay here, but then [facilitator] said ‘no, we welcome dads as well’ so … he stayed and was chatting to everyone, and I felt really comfortable (Mother, age 29, first baby) (Fox et al., 2015, p. 9).

‘Being pro‐active’: Whilst health professionals in one study felt women did not like proactive peer support (Crossland & Thomson, 2013), it was provided in six studies (Aiken & Thomson, 2013; Crossland & Thomson, 2013; Ingram, 2013;Thomson et al., 2015; Thomson, Crossland, & Dykes, 2012; Thomson, Dykes, et al., 2012).

When PSs maintained proactive contact throughout the perinatal period, some studies described their presence providing a sense of safety (Thomson et al., 2015; Thomson, Crossland, & Dykes, 2012; Thomson, Dykes, et al., 2012), meaning women gained support they might not have sought out (Thomson, Crossland, & Dykes, 2012; Thomson, Dykes, et al., 2012). Proactivity enabled important early opportunities for support (Ingram, 2013; Thomson et al., 2015; Thomson, Crossland, & Dykes, 2012), and could allow PSs to help at critical points such as when women were at risk of breastfeeding discontinuation (Ingram, 2013; Thomson, Crossland, & Dykes, 2012; Thomson, Dykes, et al., 2012):

She phoned me in the morning and that fell really well, because …I had ended up in tears the previous night. It was because I was thinking, I'm not producing milk, nothing would seem to satisfy him, winding him, changing him. I'm thinking, it must be me. So, it was really lucky when she phoned the next morning and just put my mind at ease (Mother Thomson, Crossland, & Dykes, 2012, p. 9).

‘Enabling needs‐led care’: In communities with increased social needs, proactive support was found to increase trust and enable identification of additional needs (Thomson et al., 2015; Thomson, Crossland, & Dykes, 2012; Thomson, Dykes, et al., 2012). For example, Thomson, Dykes, et al. (2012) describe a breastfeeding incentive scheme which facilitated regular face to face contact when no specific issue was at hand. This influenced the quality and depth of the peer–mother relationship:

I don't think she would have trusted me if I hadn't been seeing her so regular (peer supporter Thomson, Dykes, et al., 2012, p. 9).

PSs were enabled to access more vulnerable women and better identify women's concerns leading to closer health professional contact and increased referrals to other agencies (Thomson, Dykes, et al., 2012), a finding noted by other studies (South et al., 2012; Thomson et al., 2015; Thomson, Dykes, et al., 2012), for example:

I instantly got on to the sign language and they got lessons for her and its things like that. Fire, safety in the home, we do that, get the fire brigade round, link that in. (PS_2) (Thomson et al., 2015, p. 10).

### 3.3 Theme 3 ‘Forging partnerships with health professionals’

This theme explains the practices of ‘building trust’ and ‘collaborating via communication’ which could enable BPS services to embed within local health services. It outlines health professionals' role facilitating PSs access to mothers, and explains how PSs‐health professional partnerships could be forged.

Gatekeeping behaviour, whereby health professionals controlled PSs' access to women, was discussed by several studies; this could be passive, as when hospital (Aiken & Thomson, 2013; Dykes, 2005), or community health professionals (Curtis et al., 2007; Raine, 2003; Raine & Woodward, 2003) did not refer mothers to PSs, or active when health professionals prevented PSs accessing mothers (Aiken & Thomson, 2013; Crossland & Thomson, 2013; Curtis et al., 2007):

When the Breastfriends come into the hospital, we're the ones that go round the ward and say which ladies are breastfeeding, which ladies can they access. Because obviously we don't want them going to poorly ladies who we don't think are appropriate (Curtis et al., 2007, p. 152).

PSs sometimes attempted to circumvent gatekeeping by putting up their own posters and setting up websites (Kirkham et al., 2006), but studies described two practices that enabled access facilitation.

‘Building trust’: Trust could be built through health professionals and PSs sharing formal knowledge (Crossland & Thomson, 2013; Raine & Woodward, 2003), and PSs' ability to recognise pathological issues and, at such times, refer mothers back to health professionals (Battersby, 2001; Curtis et al., 2007; Ingram, 2013; Kirkham et al., 2006; Raine & Woodward, 2003; South et al., 2012; Thomson et al., 2015;Thomson, Crossland, et al., 2012; Thomson, Dykes, et al., 2012). For example, a health visitor explained a PSs' support of a mother with mastitis:

And the Breastfriend said (to the mother) ‘Well it's up to you, how do you feel? Ideally it would be better to carry on (breastfeeding)’. And she very much listened to the mother. Also, she referred on, and I thought that was a classic example she knew her boundary. (Annette, health professionals' focus group) (Curtis et al., 2007, p. 152).

‘Collaborating via communication’: Communication facilitated collaborative relationships (Thomson, Dykes, et al., 2012) whilst bolstering programme awareness (Thomson et al., 2015; Thomson, Crossland, et al., 2012; Thomson, Dykes, et al., 2012). It involved face‐to‐face contact with health professionals dropping into breastfeeding groups (Dykes, 2005), and telephone conversations (Thomson, Crossland, et al., 2012; Thomson, Dykes, et al., 2012). PSs used communication to convey women's needs, reveal their work, and further embed their project:

I had one on Friday that came through, went out on Friday night to see the mum, baby with tongue‐tie, referred her to tongue‐tie clinic, phoned the health visitor back which is a health visitor I had never dealt with before and told her what had happened, what I'd seen and that I had referred the lady through already and she was like ‘oh my gosh that's great have you done that, do I not have to do anything’. Sometimes, the health visitors and midwives don't know we can do stuff like that. (PSs Group Interview) (Thomson et al., 2015, p. 10).

Strong partnerships with health professionals enabled PSs' engagement with more mothers via outreach workers (Thomson et al., 2015), and by attending statutory services such as baby clinics, young mother's groups and toddler groups (Battersby, 2001; Fox et al., 2015; Ingram et al., 2005; Kirkham et al., 2006; Thomson et al., 2015). This allowed PSs to reach more vulnerable women (Thomson et al., 2015), those not necessarily planning to breastfeed (Battersby, 2001; Ingram, 2013; Thomson et al., 2015), and those from different ethnic backgrounds, enabling PSs learning about cultural differences for example:

They [Eastern European women] believe smoking is OK, but they won't smoke and breastfeed (PSs Thomson et al., 2015, p8).

## 4 DISCUSSION

This meta‐ethnography focuses on UK non‐profit BPS organisations' practices related to the context of deprivation. Findings highlight how organisations sought to make breastfeeding better known within communities, develop supportive social environments, and meet individual mothers' needs through proactive contacts. Practices fostering embedding of BPS with health professional services were important facilitators of PSs access to women. These related to effective communication and building trust between PSs and health professionals.

These practices are affirmed by wider literature which recognises the importance of community culture and the value of supportive social environments for breastfeeding mothers (i.e., Rollins et al., 2016), and the role of health professionals in facilitating women's access to peer support (i.e., Trickey et al., 2018). Our findings suggest the practices of proactive contacting and embedding with local health services enabled PSs to access a wide range of community women and learn about their needs. However, the degree to which context derived knowledge informed practices was unclear. Non‐profit organisations are theorised to be close to, and have special knowledge of, communities and therefore deliver relevant services (VCSE Review, 2018). Yet how far service commissioning involves a full cycle of needs assessment, service design, delivery, and re‐assessment, has been questioned (Rees, 2014), and the UK government's adoption, in the early 2000s, of the concept of public service markets (Sturgess, 2018) requiring organisations to bid for contracts (Rees & Mullins, 2017), must be noted. Our review found limited reference to wider social context within the available research. This may be due to social contexts rarely featuring as part of intervention development and thus becoming easily overlooked within the research/evaluation processes. Further work could be undertaken to explore whether commissioners evaluate if and how services consider and respond to social contexts as an ongoing part of usual commissioning relationships.

Health inequality has often been constructed as an issue pertaining to individuals rather than to society and wider contexts with a focus upon managing risks and behaviours rather than on social and structural factors (Blackman et al., 2012). Within organisations, evidence‐based practice and performance assessment narratives can combine and render health inequalities an issue addressed through targeted individual‐based interventions evaluated via measurable outcomes (Blackman et al., 2012). Given this policy, professional, and organisational context, it is perhaps not surprising our review reports few practices directly informed by context. We suggest future studies explore how context led service development takes place.

The finding that mothers' access to breastfeeding groups was facilitated by personal contacts (Ingram, 2013) and that groups may be attended more readily by more socially advantaged mothers (Fox et al., 2015) is supported by other literature (Hunt et al., 2021; Trickey et al., 2018). When combined with the finding that embedding services with health professionals enabled PSs better access to mothers, and that increased one to one contact resulted in more of women's needs being met, our findings suggest access is relevant in this context.

As we sought to include all studies working in areas of deprivation (defined by any measure), our search included terms for socio‐economic deprivation. All studies took place in areas of deprivation, but some defined area level deprivation by income (i.e., Curtis et al., 2007), others by broader area‐based measures (i.e., Thomson et al., 2015). While reporting of both individual participant and area level socio‐economic characteristics would have provided greater insight into participants' situations, only one paper reported individual participant socio‐economic characteristics (Graffy & Taylor, 2005). We cannot, therefore, be sure all participants were socioeconomically deprived, and ethnicity was frequently not reported. Studies did not include participants who had not engaged with peer support, meaning the acceptability of practices for these women is unknown. These limitations affected the interpretive scope of the review such that we felt unable to construct a line of argument synthesis.

This evidence synthesis has implications for research, practice and policy. It suggests future studies explore access to BPS for more vulnerable and disadvantaged groups, including the views of non‐engaged mothers, and explore how context‐based service development takes place. It suggests evaluation studies are designed to capture the contextual components of projects and how interventions interact with social contexts. Use of methodologies such realist evaluation (Pawson & Tilley, 1997) that give explicit attention to context and how intervention mechanisms operate in different social contexts may be valuable. The review highlights that key ways to improve practice concern developing positive relationships with service leads to help embed BPS and facilitate access. Policy implications include adapting formal commissioning relationships so that consideration of, and responses to, wider social context become integral.

### 4.1 Strengths and limitations

This review employed a rigorous, inclusive search strategy, a quality assurance tool, and a more critical focus on context, enabling greater understanding of how BPS services may provide support in more challenging environments. A review limitation is that LH undertook screening and selection, with decision‐making reviewed by other team members. Credibility could, perhaps, have been strengthened if two authors had worked in tandem. The use of meta‐ethnography as a review type also meant outcomes such as breastfeeding rates were not considered.

## CONFLICT OF INTEREST

All authors declare that they have no conflicts of interest.

## AUTHOR CONTRIBUTIONS

LH undertook the search and synthesis. All other authors contributed to development of the meta‐ethnography design and conduct, discussions of interpretations and to manuscript drafts.

## Supporting information


**Data S1.** Supporting information

## Data Availability

Data available on request from the authors. The data that support the findings of this study are available from the corresponding author upon reasonable request.
