# Supplementary material for: Non‐profit breastfeeding organisations' peer support provision in areas of socio‐economic deprivation in the UK: A meta‐ethnography
Source: Matern Child Nutr. 2021 Sep 30;18(1):e13271. doi: 10.1111/mcn.13271 (PMC8710092; doi:10.1111/mcn.13271)
Supplement: Supplementary file 1 — Data S1. Supporting information [file MCN-18-e13271-s001.docx]

Supplementary file 1

List of websites which were searched for the grey literature search:

[www.nct.org.uk](http://www.nct.org.uk)

[www.breastfeedingnetwork.org.uk](http://www.breastfeedingnetwork.org.uk)

<https://www.breastfeedingnetwork.org.uk/coronavirus/>

[www.laleche.org.uk](http://www.laleche.org.uk)

<http://www.llli.org/>

<https://www.laleche.org.uk/antenatal-courses/>

abm.me.uk

realbabymilk.org

[www.familiesandbabies.org.uk](http://www.familiesandbabies.org.uk)

[www.unicef.org.uk/babyfriendly](http://www.unicef.org.uk/babyfriendly)

<https://www.unicef.org.uk/babyfriendly/wp-content/uploads/sites/2/2017/07/Barriers-to-Breastfeeding-Briefing-The-Baby-Friendly-Initiative.pdf>

<https://www.unicef.org/publications/files/UNICEF_Breastfeeding_A_Mothers_Gift_for_Every_Child.pdf>

<https://www.unicef.org/topics/breastfeeding>

<https://www.evidence.nhs.uk/Search?q=breastfeeding+Peer+support+programmes>

<http://www.bmj.com/content/344/bmj.d8287>

<http://onlinelibrary.wiley.com/doi/10.1111/apa.2015.104.issue-S467/issuetoc>

<http://bmcpregnancychildbirth.biomedcentral.com/articles/10.1186/s12884-015-0581-5>

<https://www.gov.uk/government/case-studies/providing-support-and-guidance-on-breastfeeding>

<http://www.nets.nihr.ac.uk/projects/hta/131805>

<http://www.mammas.org.uk/>

<https://www.liverpoolbambis.co.uk/>

<http://www.breastfeedingsouthglos.co.uk/about-uscontact.html>

<https://gbsn.org.uk/>

<http://www.bfsupportmatters.org.uk/>

<http://blackpool.breastfeedingsupport.co.uk/>

<https://www.breastfeedinginsalford.org.uk/where-to-find-breastfeeding-support-in-salford-greater-manchester/>

<https://ukbreastfeeding.org/about/>

<http://www.babymilkaction.org/archives/16529>  

<https://www.nice.org.uk/about/what-we-do/into-practice/shared-learning-case-studies>
